# Disparities in use of skilled birth attendants and neonatal mortality rate in Guinea over two decades

**DOI:** 10.1186/s12884-021-04370-8

**Published:** 2022-01-21

**Authors:** Betregiorgis Zegeye, Bright Opoku Ahinkorah, Edward Kwabena Ameyaw, Eugene Budu, Abdul-Aziz Seidu, Comfort Z. Olorunsaiye, Sanni Yaya

**Affiliations:** 1 HaSET Maternal and Child Health Research Program, Shewarobit Field Office, Shewarobit, Ethiopia; 2 School of Public Health, Faculty of Health, University of Technology Sydney, Ultimo, Australia; 3 Department of Population and Health, University of Cape Coast, Cape Coast, Ghana; 4 College of Public Health, Medical and Veterinary Sciences, James Cook University, Townsville, Queensland Australia; 5 Department of Public Health, Arcadia University, Glenside, PA USA; 6 School of International Development and Global Studies, Faculty of Social Sciences, University of Ottawa, 120 University Private, Ottawa, ON K1N 6N5 Canada; 7 The George Institute for Global Health, Imperial College London, London, UK

**Keywords:** Skilled birth attendance, neonatal mortality, maternal health, inequality, Guinea, DHS, MICS, Global health

## Abstract

**Background:**

Maternal mortality remains high in sub-Saharan African countries, including Guinea. Skilled birth attendance (SBA) is one of the crucial interventions to avert preventable obstetric complications and related maternal deaths. However, within-country inequalities prevent a large proportion of women from receiving skilled birth attendance. Scarcity of evidence related to this exists in Guinea. Hence, this study investigated the magnitude and trends in socioeconomic and geographic-related inequalities in SBA in Guinea from 1999 to 2016 and neonatal mortality rate (NMR) between 1999 and 2012.

**Methods:**

We derived data from three Guinea Demographic and Health Surveys (1999, 2005 and 2012) and one Guinea Multiple Indicator Cluster Survey (2016). For analysis, we used the 2019 updated WHO Health Equity Assessment Toolkit (HEAT). We analyzed inequalities in SBA and NMR using Population Attributable Risk (PAR), Population Attributable Fraction (PAF), Difference (D) and Ratio (R). These summary measures were computed for four equity stratifiers: wealth, education, place of residence and subnational region. We computed 95% Uncertainty Intervals (UI) for each point estimate to show whether or not observed SBA inequalities and NMR are statistically significant and whether or not disparities changed significantly over time.

**Results:**

A total of 14,402 for SBA and 39,348 participants for NMR were involved. Profound socioeconomic- and geographic-related inequalities in SBA were found favoring the rich (PAR = 33.27; 95% UI: 29.85–36.68), educated (PAR = 48.38; 95% UI: 46.49–50.28), urban residents (D = 47.03; 95% UI: 42.33–51.72) and regions such as Conakry (R = 3.16; 95% UI: 2.31–4.00). Moreover, wealth-driven (PAF = -21.4; 95% UI: −26.1, −16.7), education-related (PAR = -16.7; 95% UI: −19.2, −14.3), urban-rural (PAF = -11.3; 95% UI: −14.8, −7.9), subnational region (R = 2.0, 95% UI: 1.2, 2.9) and sex-based (D = 12.1, 95% UI; 3.2, 20.9) inequalities in NMR were observed between 1999 and 2012. Though the pattern of inequality in SBA varied based on summary measures, both socioeconomic and geographic-related inequalities decreased over time.

**Conclusions:**

Disproportionate inequalities in SBA and NMR exist among disadvantaged women such as the poor, uneducated, rural residents, and women from regions like Mamou region. Hence, empowering women through education and economic resources, as well as prioritizing SBA for these disadvantaged groups could be key steps toward ensuring equitable SBA, reduction of NMR and advancing the health equity agenda of “no one left behind.”

## Background

Maternal and child health promotion and reduction of mortality are key components of the global maternal health and developmental agenda, as reflected in the Millennium Development Goals (MDGs) and currently in the Sustainable Development Goals (SDGs) agenda for 2030 [1, 2]. Despite a global decrease, maternal mortality is still a major public health problem [3]. Worldwide, approximately 800 women die each day because of complications during pregnancy, childbirth, and puerperium [4]. Sub-Saharan Africa (SSA) accounts for 68% of daily maternal deaths, which is approximately 533 maternal deaths per 100,000 live births, or 200,000 maternal deaths a year [4]. In Guinea, though considerable improvements have occurred, it is still high with a maternal mortality ratio (MMR) of 550 per 100,000 live births in 2016 [5].

Majority of maternal and perinatal deaths are caused by obstetric complications. A substantial proportion of these are avoidable when women can make use of the available health care offered by skilled health care providers [6–9]. Giving birth with the help of skilled birth attendants (SBAs) will assuage maternal and perinatal mortality by averting or early management of most obstetric complications [10]. Globally, some of the dominant causes of neonatal mortality are intrapartum-related deaths from birth asphyxia or preterm birth [11]. Between the years 1990 and 2016, neonatal mortality rates (NMRs) declined (i.e., from 37 to 19 deaths per 1000 live births). However, about 7000 neonatal deaths occur daily [12]. Across countries and regions, there is unequal distribution of neonatal deaths [2]. The two leading regions that accounted for about 80% of neonatal deaths in 2016 are Southern Asia (39%) and SSA (38%) [13]. In Guinea, about 31 babies die daily prior to their first month and 27 stillbirths occur every day [14, 15]. Tremendous decline in neonatal mortality can occur through SBA, because that prevents 40–70% of newborn deaths [16–19].

The World Health Organization (WHO) conceives SBAs as competent healthcare providers (e.g., doctors, midwives, or nurses) with training in how to handle uncomplicated pregnancies effectively as well as births and the postpartum period. They also identify, manage, and refer obstetric and neonatal complications [20, 21].

Guinea is one of the poorest countries in the world [22]. For instance, Guinea was rated 182 of 188 countries by the 2015 Human Development Index (HDI) [22]. Per capita growth is also very low and averaged 0.6% percent in 1998–2016. The country has about 2.6 average years of schooling and about 70% of girls are enrolled in primary level of education against 81% for boys. Enrollments in secondary schools stand at 24% (females) and 37% (males). About six out of ten households without education are about three times poorer than households with highly educated heads [22]. Generally, there is limited access to essential services with only 28% having access to electricity and 20% having improved sanitation [22].

On existing social and financial obstacles, there is scarcity in equipment, infrastructure, and human resources and the few available are distributed unequally [23]. A large proportion of girls in Guinea give birth by their 17th birthday and there is inadequacy of maternal health care [23]. Out of pocket payment is prevalent and this constitutes a great burden to less endowed households [24]. Generally, there is inequity in public health spending as this seems to align towards Conakry, where huge numbers of health personnel practice [24]. Rural dwellers are deprived in terms of available basic care [24] and only 40% of women across rural regions obtain four (or more) antenatal health consultations relative to 71% in urban locations [24].

Globally, there have been improvements in the utilization of SBAs from 71% in 2013 to 81% in 2019 [25]. Significant inequalities, however, exist across regions, with countries in West and Central Africa recording just 4% rise in coverage from 55% in 2013 to 59% in 2019 [25]. Coverage of SBAs in SSA is lower compared to all other regions in the world [25]. Narrowing these inequalities is foundational to the new ‘leaving no one behind’ slogan or strategic framework for action in the SDG regime [26, 27]. There are appreciable policies that are related to reduction of inequality in maternal healthcare in Guinea. For instance, in 2010, the Ministry of Health (MoH) of Guinea introduced a free emergency obstetric care policy in all public health facilities [28].

However, there is a dearth of evidence in Guinea about the magnitude and over-time dynamics of socioeconomic and geographic disparities in SBA and NMR. Hence, this study aimed to answer two major questions. First, what are the trends in coverage of SBA and NMR across socio-economic, urban-rural, and subnational subpopulations in Guinea from 1999 to 2016? Second, what is the magnitude of socio-economic, urban-rural, and subnational regional disparities in SBA and NMR in Guinea from 1999 to 2016?

## Methods

### Data source

We extracted data from three waves of the Guinea Demographic and Health Surveys (DHS: 1999, 2005, 2012) and one wave of the Guinea Multiple Indicator Cluster Survey (MICS) (2016) which are all deposited in the WHO Health Equity Assessment Toolkit (HEAT) software. Both DHS and MICS collect nationally representative, wide-ranging data related to health, education and mortality which can be used for tracking progress toward the MDGs and SDGs [29, 30]. The Guinea DHS is conducted by the National Institute of Statistics of the Ministry of Planning with financial support from the United States Agency for International Development (USAID) and technical assistance from Inner-City Fund (ICF) International, based in the USA. MICS is, however, conducted with technical assistance of United Nations Children’s Fund (UNICEF) [29, 30].

For all DHS and MICS surveys, a two-stage stratified cluster sampling technique was used. This is targeted to provide adequate representation of urban and rural settings as well as the eight domains corresponding to the five administrative regions in Guinea in 1999 (i.e., Lower Guinea, Central Guinea, Upper Guinea, Forest Guinea and Conakry) and eight regions for the rest three years of surveys (2005, 2012 and 2016) (i.e., Boké, Conakry, Faranah, Kankan, Kindia, Labé, Mamou and N’Zérékoré), for which we have an estimate for all key indicators [29, 30]. First, clusters or enumeration areas were drawn across the entire national territory from the list of enumeration areas established in the most recent census that preceded each of the surveys. Clusters were selected using probability proportional to size (PPS) [29, 30]. Thereafter, in the second stage, in each cluster, fixed numbers of 28–30 households were selected using systematic random sampling technique, and in each household, women of reproductive age (15–49 years), who had spent the last night in the household were eligible for the survey [29, 30].

### Measures

The outcome variables, for which we assessed inequality, were SBA and NMR. SBA refers to births that were assisted by skilled health personnel [25]. This outcome variable was dichotomized and coded based on the assistance at birth (SBA =1, unskilled attendant = 0) [25]. We considered the percentage of SBA in the two or three years preceding the survey).

Inequality was also measured for NMR and refers to the number of deaths during the first 28 completed days of life per 1000 live births in a given year or another period [31]. The birth histories data in the DHS has information on the birth dates and age of death of neonates. The analyses involved data on live births that occurred 5 years prior to the surveys [31].

SBA inequality was measured using four equity stratifiers: economic status, educational status, place of residence and subnational region. Economic status was examined using wealth index, which is calculated based on characteristics and assets of the household. In DHS, wealth index is calculated using Principal Component Analysis (PCA) [32]. It is coded as poorest, poorer, middle, richer, and richest quintiles. Educational status of the mother was categorized as no education, primary school, and secondary or higher education. Place of residence was categorized as urban or rural. Subnational region was grouped into five regions for the 1999 survey; Lower Guinea, Central Guinea, Upper Guinea, Forest Guinea, and Conakry and eight regions (Boke, Conakry, Faranah, Kankan, Kindia, Labe, Mamou, Nzerekore) for the other surveys. In addition to that, NMR was examined using neonate sex and coded as male or female. Since the 2016 NMR data were not available in the HEAT software, the analysis was limited to 1999–2012.

### Statistical analyses

The WHO’s HEAT application promotes the conduct of disaggregated analysis [33, 34]. Analyses were carried out using two major steps. First, coverage of SBA was disaggregated using four inequality dimensions. Then, absolute, and relative socioeconomic and geographic inequalities were calculated using four summary measures, namely Difference (D), Population Attributable Fraction (PAF), Population Attributable Risk (PAR) and Ratio (R). The complete procedures for calculation and interpretation of the summary measures have been discussed elsewhere [35]. For favorable health indicators (indicators that include healthcare such as SBA), positive values of the summary measures indicate uneven higher coverage among advantaged subgroups while higher absolute values indicate higher degrees of inequality. Zero values indicate the absence of inequality. R and D are simple summary measures. However, PAR and PAF are weighted and complex measures that account for all subgroups under the inequality dimension, producing robust estimations of inequality among all populations [33–35]. Unlike complex measures, simple measures do not possess characteristics [33, 35]. However, straightforward calculation and interpretation could be applied in simple measures. WHO recommends combination of both methods for comprehensive investigation and assessing results from various perspectives [33, 35].

More specifically Differences were calculated by subtracting SBA coverage in the poorest quintile for economic status and non-educated for education status from the richest quintile for economic status and secondary schools and above for education status. In the same way, for place of residence, Differences were calculated by subtracting urban from rural residents, and for the subnational region, the region with highest SBA coverage minus the region with lowest SBA coverage.

Ratio was calculated by dividing the two subgroups mentioned for each dimension to render relative values. Inequality did not exist if D had a value of 0 or R had a value of 1. PAR was also calculated by subtracting SBA coverage of the national average from the reference group.

Reference groups for economic status, education status and place of residence were richest, secondary school and above and urban residence. For subnational region, the reference group was the region with the highest SBA estimate, Conakry. PAF was calculated by dividing PAR by the national average and multiplying by 100 [33, 35].

Regarding NMR, we calculated D as NMR in the “non-educated” subgroup and deducted NMR in “secondary education and above” subgroup. In the case of economic status, we computed it as NMR within the poorest subgroup minus NMR within the richest subgroup. In addition, D was computed as NMR within the rural population minus NMR within the urban populations in relation to place of residence. Besides, the region bearing the lowest estimate was deducted from the highest estimate region. R was gauged with the same subgroups. The estimated national NMR was subtracted from the NMR for the reference subgroup in computing the PAR. In the case of the ordered aspect, yref denoted the most-advantaged sub-group and this represents research participants with at least second cycle education. In the same way, the wealthiest sub-group was used for the economic status and sub-groups with the least estimates were utilized for binary variables such as sex (females) and residence (urban). With respect to the non-ordered variables (e.g., subnational region), the reference group denoted the region having the least estimate and we calculated PAF by dividing PAR by the national average and multiplying by 100.

PAR or PAF values of zero indicated absence of inequality and greater absolute values of both complex measures indicated higher inequality. Each point estimate had an Uncertainty Interval (95% UI) to assess SBA inequalities that were statistically significant. Inequality trends in SBA were assessed in caution and by referring to the UI of each summary measure of different surveys. That means, if the UI does not overlap, there was increasing or decreasing changes, but overlapping of UIs was considered as constant pattern. However, small, and large overlapping UI was not treated equally, and authors considered this important concept during interpretations of trends. Findings were presented per recommendations of the Strengthening Reporting of Observational studies in Epidemiology (STROBE) [36].

## Results

**Fig. 1:**
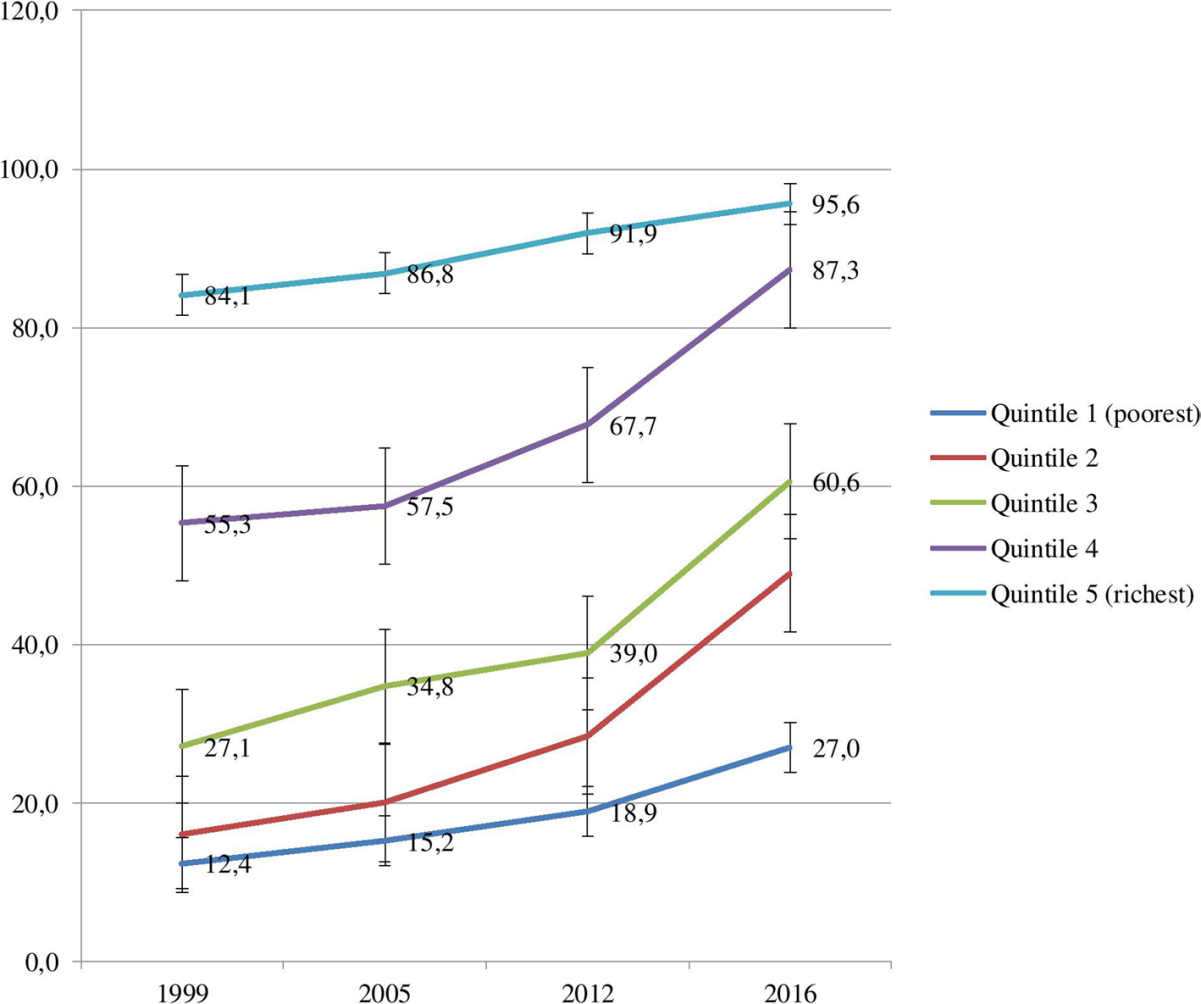
SBA coverage in Guinea by wealth quintiles: Evidence from GDHS (1999–2012) and GMICS (2016)

A total of 14,402 participants for SBA and 39,348 for NMR were involved in this study. Out of the SBA sample, 10,514 women (73.0%) were rural residents. Regarding maternal educational level, 11,554 (80.2%) had no formal education, only 1565 (10.9%) had primary school and 1282 (8.9%) had secondary or higher education. Figure 1 shows trends in the coverage of SBA among socioeconomic subgroups from 1999 to 2016. Across wealth quintiles, in the first and last surveys, SBA coverage among the richest wealth quintile 5 was higher by 71.7 (in 1999) and 68.5 (in 2016) percentage points as compared to the poorest wealth quintile 1. SBA coverage improved across wealth quintiles, albeit not proportionally in the 17 years of survey periods. For example, SBA coverage among the poorer wealth quintile 2 increased by 33 percentage points, by 33.6 in the middle wealth quintile 3 and by 32.0 among the richer wealth quintile 4 from 1999 to 2016 as compared with 14.6 among the poorest wealth quintile 1 and 11.5 among the richest wealth quintile 5.

**Fig. 2:**
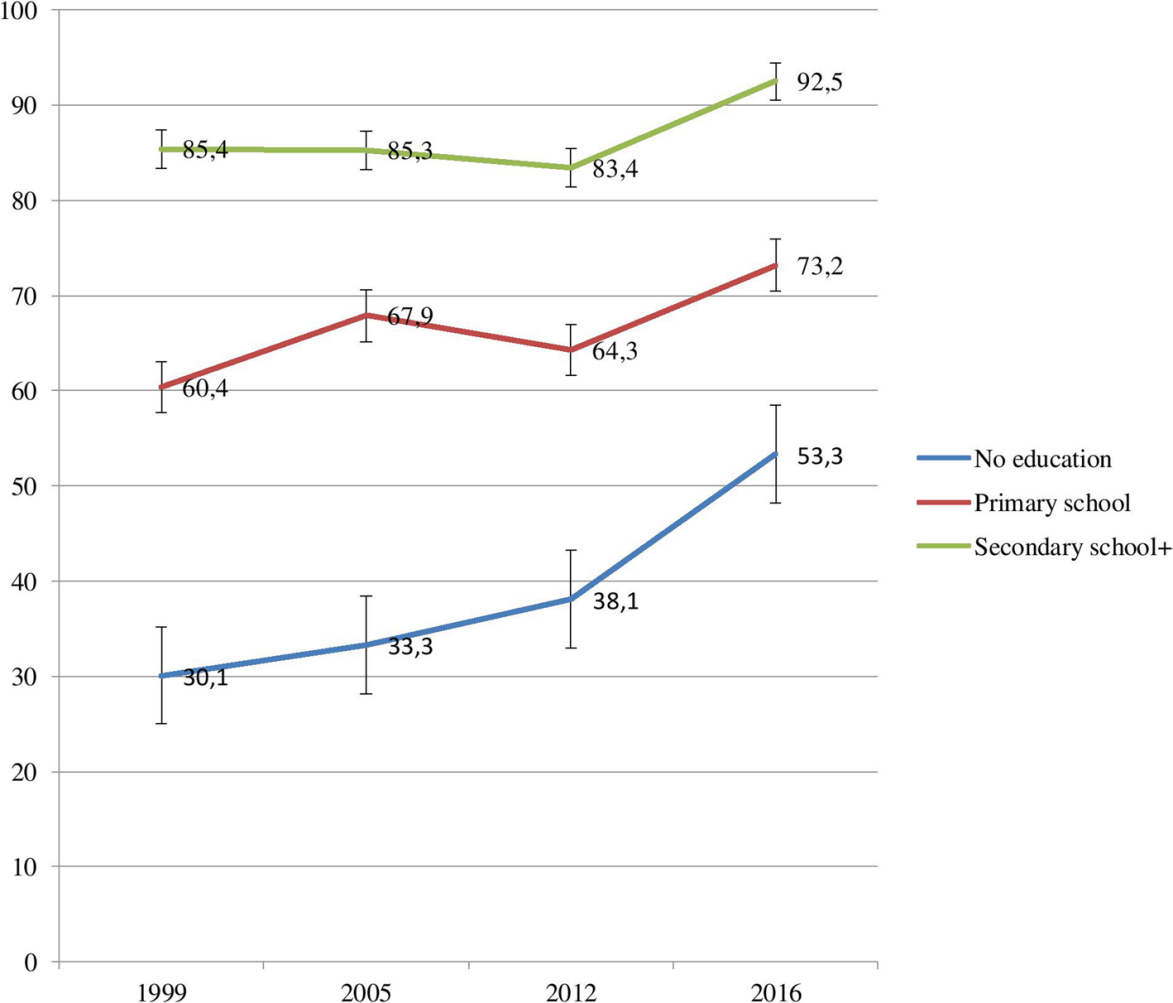
SBA coverage in Guinea by educational status: Evidence from GDHS (1999–2012) and GMICS (2016)

SBA coverage also differed across educational subgroups. SBA coverage among those with secondary or higher education was higher by 55.3 percentage points in 1999 and 39.1 percentage points in 2016 as compared to those with no formal education (Fig. 2). SBA coverage increased by 23.2 percentage points among subgroups without formal education whereas it only increased by 7.1 percentage points among those with secondary or higher education subgroups from 1999 to 2016.

**Fig. 3:**
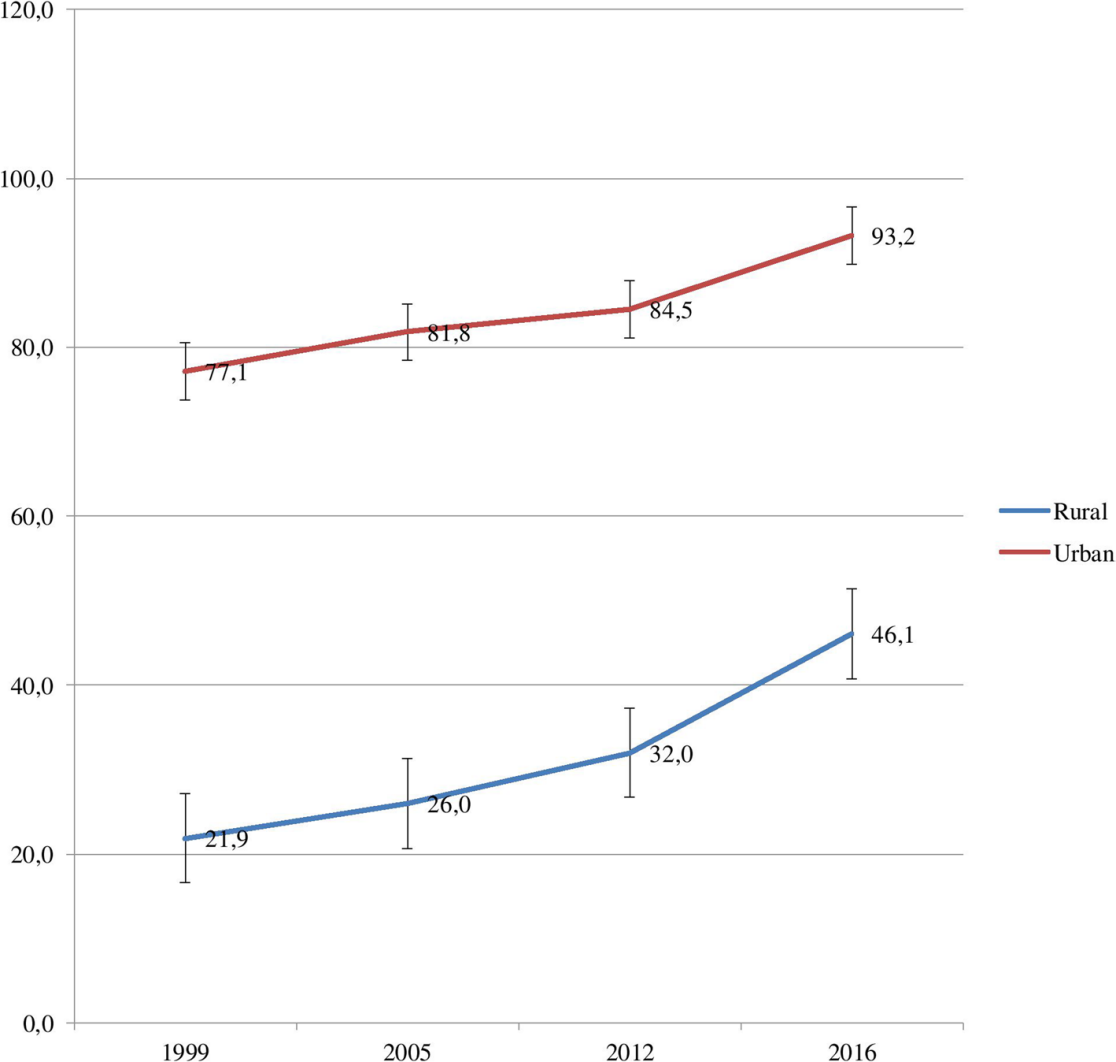
SBA coverage in Guinea by place of residence: Evidence from GDHS (1999–2012) and GMICS (2016)

Similarly, a 47.0 percentage-point difference was observed between urban and rural subgroups in the 2016 survey. SBA coverage increased by 24.3 percentage points among rural subgroups while it increased only by 16.1 percentage points among urban subgroups between 1999 and 2016 (Fig. 3).

Regarding coverage across regions, our findings indicate that improvements varied substantially over the 11-year period from 2005 to 2016 across subnational regions in Guinea. For example, SBA coverage increased by 45.1 percentage points in Boke region, and by 11.3 percentage points in Mamou region. As shown in Table 1, SBA coverage in Guinea was 35.8% in 1999, 38.9% in 2005, 46.3% in 2012, and 62.3% in 2016 (Table 1).

**Table 1:** SBA coverage across socioeconomic and geographic subpopulations in Guinea: Evidence from Guinea DHSs (1999–2012) and MICS (2016)

Dimension of Inequality	Subgroup	1999			2005		2012		2016	
		Estimate (95% UI)		Pop^**n**^	Estimate (95% UI)	Pop^**n**^	Estimate (95% UI)	Pop^**n**^	Estimate (95% UI)	Pop^**n**^
Economic status	Quintile 1 (poorest)	12.4 (9.3–16.4)		801	15.2 (10.6–21.3)	955	18.9 (14.6–24.2)	945	27.0 (22.5–32.1)	567
	Quintile 2	16.0 (12.5–20.3)		727	20.0 (16.7–23.8)	857	28.4 (23.8–33.4)	907	49.0 (42.7–55.3)	620
	Quintile 3	27.1 (22.9–31.8)		691	34.8 (29.1–41.1)	825	39.0 (33.0–45.4)	880	60.6 (54.3–66.6)	570
	Quintile 4	55.3 (49.3–61.1)		658	57.5 (52.7–62.2)	725	67.7 (62.7–72.4)	824	87.3 (83.1–90.5)	564
	Quintile 5 (richest)	84.1 (80.6–87.1)		547	86.8 (82.5–90.2)	598	91.9 (88.0–94.5)	679	95.6 (92.9–97.3)	456
Education	No education	30.1 (27.1–33.2)		2941	33.3 (29.9–36.9)	3421	38.1 (34.3–42.1)	3243	53.3 (49.7–56.9)	1949
	Primary school	60.4 (53.3–67.2)		287	67.9 (61.8–73.5)	340	64.3 (58.9–69.4)	546	73.2 (67.5–78.2)	392
	Secondary school+	85.4 (78.8–90.2)		198	85.3 (78.7–90.0)	199	83.4 (78.1–87.7)	447	92.5 (88.4–95.2)	438
Place of residence	Rural	21.9 (18.8–25.22)		2558	26.0 (22.5–29.9)	3048	32.0 (27.5–36.9)	3085	46.1 (42.1–50.2)	1823
	Urban	77.1 (72.6–81.0)		867	81.8 (77.3–85.5)	913	84.5 (81.2–87.3)	1152	93.2 (90.4–95.1)	956
Subnational region	Lower Guinea	Boke	31.8 (25.2–39.1)	703	26.4 (20.5–33.4)	454	42.9 (33.2–53.2)	392	71.6 (62.9–78.8)	326
	Central Guinea	Conakry	18.5 (15.0–22.6)	711	90.1 (85.1–93.6)	422	90.1 (85.4–93.4)	626	95.6 (92.3–97.5)	494
	Upper Guinea	Faranah	20.7 (15.7–26.7)	663	33.5 (26.4–41.6)	339	29.8 (22.4–38.5)	436	46.5 (36.8–56.4)	239
	Forest Guinea	Kankan	39.0 (32.4–46.0)	909	41.1 (32.7–50.2)	616	45.0 (34.8–55.6)	783	53.2 (45.9–60.4)	549
	Conakry	Kindia	87.1 (83.0–90.3)	437	28.2 (22.0–35.3)	563	41.8 (31.9–52.4)	597	64.4 (57.3–70.8)	361
	NA	Labe	NA	NA	18.8 (14.1–24.5)	391	32.2 (24.6–40.9)	385	45.6 (34.8–56.8)	219
	NA	Mamou	NA	NA	18.9 (13.6–25.7)	263	19.6 (14.8–25.5)	275	30.3 (22.8–38.8)	218
	NA	Nzerekore	NA	NA	42.8 (32.7–53.6)	911	43.0 (32.3–54.5)	741	60.2 (48.7–70.7)	370
National average			35.8%		38.9%		46.3%		62.3%	

### NMR distribution across different subpopulation

NMR in 1999 was 51.8 per 1000 live births, 54.8 in 2005 and 40.4 in 2012 (Table 2). NMR in the poorest wealth quintile 1 in 1999 was higher by 17.6 per 1000 live births, by 21.7 in 2005 and by 17.2 in 2012 as compared with the richest wealth quintile 5. NMR in 1999 among mothers who had no formal education was higher by 29.6 per 1000 live births, by 13.5 in 2005 and by 17.9 in 2012, compared with mothers who had attended secondary school or higher levels of education. NMR in 1999 among rural residents was higher by 13.7 per 1000 live births, by 11.7 in 2005 and by 6.1 in 2012 compared with urban residents. NMR in 1999 among male neonates was higher by 15.2 per 1000 live births, by 17.3 in 2005 and by 12.1 in 2012 compared with female neonates. NMR in 2005 among Faranah region was higher by 35.4 per 1000 live births compared with Conakry region, and in 2012 higher by 25.9 per 1000 live births among Kankan region compared with Nzerekore region (Table 2).

**Table 2:** Neonatal mortality rate across socioeconomic and geographic subpopulations in Guinea: Evidence from Guinea DHSs (1999–2012)

Dimension of Inequality	Subgroup		1999		2005		2012	
			Estimate (95% UI)	Pop^**n**^	Estimate (95% UI)	Pop^**n**^	Estimate (95% UI)	Pop^**n**^
Economic status	Quintile 1 (poorest)		56.1 (47.1–66.7)	2941	60.0 (48.9–73.3)	3238	49.0 (39.8–60.1)	3190
	Quintile 2		57.9 (47.2–70.9)	2529	60.9 (51.9–71.3)	2851	37.1 (28.7–47.7)	3125
	Quintile 3		54.1 (44.8–65.1)	2369	61.3 (51.8–72.3)	2820	40.5 (33.2–49.5)	2949
	Quintile 4		48.6 (39.8–59.3)	2304	46.6 (38.2–56.8)	2469	40.4 (30.4–53.5)	2642
	Quintile 5 (richest)		38.5 (30.2–48.9)	1944	38.2 (27.3–53.2)	1957	31.7 (22.7–44.3)	2017
Education	No education		54.6 (49.1–60.6)	10,516	55.7 (50.4–61.4)	11,703	41.6 (36.6–47.2)	11,348
	Primary school		40.4 (27.6–58.6)	870	52.2 (38.5–70.4)	1017	43.5 (29.1–64.4)	1489
	Secondary school+		24.9 (15.0–41.1)	702	42.1 (25.6–68.5)	616	23.6 (15.2–36.4)	1085
Place of residence	Rural		55.3 (49.1–62.1)	9072	57.4 (52.1–63.4)	10,302	41.9 (36.5–48.1)	10,443
	Urban		41.5 (34.8–49.4)	3017	45.7 (36.8–56.5)	3034	35.8 (28.2–45.3)	3479
Sex	Female		44.0 (38.2–50.6)	5865	45.8 (39.8–52.8)	6464	34.2 (28.7–40.7)	6816
	Male		59.2 (52.7–66.5)	6224	63.2 (56.2–70.9)	6872	46.3 (40.2–53.3)	7107
Subnational region	Lower Guinea	Boke	44.9 (35.8–56.2)	2557	46.7 (36.6–59.3)	1586	43.0 (29.2–62.8)	1322
	Central Guinea	Conakry	47.4 (37.7–59.5)	2760	37.0 (24.0–56.4)	1345	32.8 (21.9–48.8)	1798
	Upper Guinea	Faranah	61.7 (51.1–74.5)	2227	72.4 (56.7–91.9)	1136	44.6 (31.1–63.7)	1478
	Forest Guinea	Kankan	58.8 (47.3–72.8)	3020	49.7 (41.8–59.1)	2005	50.2 (38.3–65.4)	2495
	Conakry	Kindia	43.1 (34.2–54.2)	1524	61.5 (50.8–74.2)	1947	40.0 (29.2–54.5)	2107
	NA	Labe	NA	NA	48.4 (35.6–65.4)	1344	48.2 (35.3–65.6)	1296
	NA	Mamou	NA	NA	54.8 (45.4–65.9)	913	49.7 (38.0–64.7)	1008
	NA	Nzerekore	NA	NA	62.1 (50.1–76.7)	3056	24.2 (17.6–33.2)	2414
National average			51.8	12,089	54.8	13,336	40.4	13,923

Notes: NA not applicable for 1999 due to the subnational region in 1999 were five; *UI* Uncertainty Interval, *Pop*^*n*^ Population

### Magnitude and trends in inequalities in SBA

We found significant absolute and relative wealth-driven inequality in SBA in Guinea from 1999 to 2016 using simple (D, R) and complex (PAR, PAF) measures. For example, the D measure (68.5, 95% UI 63.3–73.8) in the 2016 survey indicate that there is an absolute wealth-driven inequality in SBA. In addition, it also indicates that SBA coverage was higher by 68.5 percentage points among the richest quintile as compared to the poorest quintile. Similarly, the PAF measure (53.4, 95% UI 47.9–58.9) in the 2016 survey indicates pro-rich relative wealth-driven inequality in SBA. It also indicates the 2016 national SBA coverage could be improved by 53.4 percentage points (95% UI 47.9–58.9%), if the country avoided the relative economic-related inequality. Except for decreasing patterns from 2012 to 2016 by PAR measures, patterns of absolute economic inequality were constant overtime. The pattern of relative economic inequality, however, decreased by complex measure (PAF) and was constant by simple measure (R) over the 17 years survey periods. Significant absolute and relative education-related inequalities were observed both by simple and complex measures from 1999 to 2016. For instance, PAR measure (30.2, 95% UI 28.9–31.3) in 2016 indicates absolute education-related inequality in SBA that favored women with secondary education. It further shows that the 2016 national SBA coverage could be improved by 30.2 percentage points if the country could avoid absolute education-related disparities in the country. Additionally, the R measure 1.7 (95% UI 1.6–1.9) in the same survey indicates significant relative education-based inequality in SBA. It also indicates that SBA coverage among subgroups with secondary or higher education was 1.7 times higher compared to non-educated subgroups in 2016. Decreasing patterns of both types of inequalities were seen over the 17 years survey periods by complex measures (PAR, PAF). Simple measures (D, R) indicated constant education-based inequality patterns, except a decreasing R measure from 2012 to 2016.

Furthermore, we found profound absolute and relative urban-rural inequalities in SBA both by simple and complex measures from 1999 to 2016. For instance, the PAF measure (49.5, 95% UI 47.3–51.7) in 2016 survey indicates significant pro-urban relative inequality in SBA. It also shows the country could improve the 2016 SBA coverage by approximately 50% if Guinea could avoid urban-rural relative inequality. Likewise, the D measure 47 (95% UI 42.3–51.7) in the same survey confirmed absolute urban-rural inequality in SBA in which SBA coverage among urban residents was higher by 47 percentage points compared to rural residents (Table 3). Decreasing patterns of absolute and relative inequalities were seen over the 17 years survey periods by the complex measures. Simple measures (D, R) indicate constant urban-rural inequality patterns except a decreasing R measure from 2012 to 2016.

**Table 3:** Magnitude and trends in inequalities in SBA in Guinea: Evidence from Guinea DHSs (1999–2012) and MICS (2016)

Dimension		1999	2005	2012	2016
	Measure	Estimate (95% UI)	Estimate (95% UI)	Estimate (95% UI)	Estimate (95% UI)
Household economic status	D	71.7 (66.9–76.4)	71.7 (65.2–78.2)	72.9 (67.2–78.6)	68.5 (63.3–73.8)
	PAF	134.7 (128.6–140.8)	123.3 (117.8–128.9)	98.4 (93.3–103.5)	53.4 (47.9–58.9)
	PAR	48.3 (46.1–50.4)	48.0 (45.8–50.1)	45.6 (43.2–47.9)	33.3 (29.9–36.7)
	R	6.8 (4.9–8.7)	5.7 (3.7–7.7)	4.9 (3.6–6.1)	3.5 (2.9–4.2)
Maternal educational level	D	55.3 (49.0–61.6)	52.0 (45.4–58.6)	45.3 (39.14–51.45)	39.1 (34.22–44.02)
	PAF	138.3 (136.1–140.4)	119.3 (117.5–121.1)	80.2 (78.3–82.1)	48.4 (46.5–50.3)
	PAR	49.6 (48.8–50.3)	46.4 (45.7–47.1)	37.1 (36.2–38.0)	30.2 (29.0–31.3)
	R	2.8 (2.5–3.2)	2.6 (2.2–2.9)	2.2 (1.9–2.4)	1.7 (1.6–1.9)
Place of residence	D	55.2 (50.0–60.4)	55.7 (50.3–61.2)	52.5 (46.9–58.1)	47.0 (42.3–51.7)
	PAF	115.0 (112.0–118.1)	110.3 (107.8–112.8)	82.5 (80.3–84.7)	49.5 (47.3–51.7)
	PAR	41.2 (40.1–42.3)	42.9 (41.9–43.9)	38.2 (37.2–39.2)	30.8 (29.5–32.2)
	R	3.5 (3.0–4.1)	3.14 (2.67–3.61)	2.6 (2.2–3.0)	2.0 (1.8–2.2)
Subnational region	D	68.6 (63.4–73.8)	71.3 (64.7–78.0)	70.5 (63.9–77.2)	65.4 (57.0–73.8)
	PAF	142.9 (135.5–150.4)	131.7 (122.0–141.4)	94.6 (84.6–104.6)	53.4 (43.9–63.0)
	PAR	51.2 (48.5–53.9)	51.2 (47.4–55.0)	43.8 (39.2–48.4)	33.3 (27.3–39.3)
	R	4.71 (3.7–5.7)	4.80 (3.5–6.1)	4.6 (3.3–5.9)	3.2 (2.31–4.0)

Moreover, we found considerable absolute and relative regional inequalities in SBA from 1999 to 2016 by all four measures. For example, the PAR measure of 33.3 (95% UI27.3–39.3) in the 2016 survey indicates significant absolute regional inequalities in SBA and also signifies the possibility of improvement of the 2016 SBA coverage by 33.3 percentage points, if the country could avoid absolute region-based inequality. The R measure of 3.2 (95% UI 2.3–4.0) in the same survey specified the presence of relative regional inequality in SBA. It also showed that SBA coverage in the Conakry region (with highest SBA-coverage) was 3.2 times higher compared to the Mamou region (with lowest SBA-coverage). While decreasing regional patterns were observed by complex measures over the 17 years survey period, constant patterns were observed by simple measures (Table 3).

### Magnitude and trends in NMR

Table 4 shows absolute and relative socioeconomic, gender and geographic inequalities in NMR between 1999 and 2012 with more burden among disadvantaged subpopulations. More specifically, the study shows wealth-driven inequality in NMR with more concentration among economically underprivileged subpopulations with both complex (PAR, PAF) and simple (D) measures. For instance, the PAF measure in 1999 (−25.7, 95% UI: −29.6, −21.9), 2005 (−30.2, 95% UI: −33.8, −26.5) and 2012 (−21.4, 95% UI: −26.1, −16.7) indicating relative economic-related inequality with more burden among poorest subpopulation and inequality was more widened in 2005. The 1999, 2005 and 2012 national average NMR could be reduced by approximately 26, 30 and 21 newborns per 1000 live births respectively if the country could avoid the relative wealth-driven inequality.

**Table 4:** Magnitude and trends in inequalities in neonatal mortality rate in Guinea: Evidence from Guinea DHSs (1999–2012)

Dimension		1999	2005	2012
	Measure	Estimate (95% UI)	Estimate (95% UI)	Estimate (95% UI)
Household economic status	D	17.6 (4.1, 31.1)	21.7 (4.1, 39.3)	17.2 (2.5, 31.8)
	PAF	−25.7 (−29.6, −21.9)	−30.2 (−33.8, −26.5)	−21.4 (−26.1, −16.7)
	PAR	−13.3 (−15.3, −11.3)	−16.5 (−18.5, −14.5)	−8.6 (−10.5, −6.7)
	R	1.4 (1.0, 1.8)	1.5 (0.9, 2.1)	1.5 (0.9, 2.1)
Maternal educational level	D	29.6 (15.8, 43.4)	13.5 (−7.8, 34.9)	17.9 (6.4, 29.5)
	PAF	−51.8 (−57.9, −45.7)	−23.0 (−30.0, −16.1)	−41.4 (−47.5, −35.4)
	PAR	−26.8 (−30.0, −23.7)	−12.6 (−16.4, −8.8)	−16.7 (−19.2, −14.3)
	R	2.1 (1.0, 3.3)	1.3 (0.6, 1.9)	1.7 (0.9, 2.5)
Place of residence	D	13.7 (4.0, 23.4)	11.7 (0.5, 23.0)	6.1 (−4.1, 16.4)
	PAF	−19.9 (−22.8, −16.9)	−16.6 (−19.4, −13.7)	−11.3 (−14.8, −7.9)
	PAR	−10.3 (−11.8, −8.8)	−9.1 (−10.6, −7.5)	−4.6 (−6.0, −3.2)
	R	1.3 (1.0, 1.6)	1.2 (0.9, 1.5)	1.1 (0.8, 1.4)
Neonate sex	D	15.2 (5.9, 24.4)	17.3 (7.5, 27.0)	12.1 (3.2, 20.9)
	PAF	−15.1 (−16.8, −13.3)	−16.2 (−17.8, −14.6)	−15.3 (−17.3, −13.2)
	PAR	−7.8 (−8.7, −6.9)	−8.9 (−9.7, −8.0)	−6.1 (−7.0, −5.3)
	R	1.3 (1.1, 1.5)	1.3 (1.1, 1.6)	1.3 (1.0, 1.6)
Subnational region	D	18.6 (3.3, 33.8)	35.4 (11.8, 58.9)	25.9 (10.4, 41.3)
	PAF	−16.7 (−21.2, −12.2)	−32.4 (−36.9, −27.9)	−39.9 (−43.8, −35.9)
	PAR	−8.6 (−11.0, −6.3)	−17.8 (−20.2, −15.3)	−16.1 (−17.7, −14.5)
	R	1.4 (1.0, 1.8)	1.9 (0.9, 2.9)	2.0 (1.2, 2.9)

Notes: *D* Difference, *PAF* Population Attributable Fraction, *PAR* Population Attributable Risk, *R* Ratio

Both absolute and relative education-related inequality in NMR was observed between 1999 and 2012 using simple (D) and complex (PAR, PAF) measures with more burden for mothers who had not attended formal education. For instance, the PAR measures −26.8 (95% UI: −30.0, −23.7) in 1999, −12.6 (95% UI: −16.4, −8.8) in 2005 and − 16.7 (95% UI: −19.2, −14.3) in 2012 indicated absolute education-related inequality with more concentration among neonates of mothers who did not attend formal education.

The study also shows pro-urban absolute and relative inequality in NMR between 1999 and 2012, but with a decreasing pattern. For instance, the PAF measure −19.9 (95% UI: −22.8, −16.9) in 1999, −16.6 (95% UI: −19.4, −13.7) in 2005 and − 11.3 (95% UI: −14.8, −7.9), in 2012 indicated a relative place of residence inequality in NMR with more burden among rural residents. Moreover, we found gender-based absolute and relative inequality in NMR between 1999 and 2012 with more concentration among male neonates. We found absolute and relative subnational region inequality in NMR between 1999 and 2012 with increasing pattern in relative inequality and mixed pattern in absolute inequality. For instance, the PAF measure −39.9 (95% UI: −43.8, −35.9) and PAR measure −16.1 (95% UI: −17.7, −14.5) in 2012 confirmed absolute and relative subnational region inequality. Moreover, it shows the country could reduce the 2012 national average of NMR by approximately 40 and 16 newborns per 1000 live births if the country could avoid relative and absolute subnational region inequality (Table 4).

## Discussion

The national average of SBA coverage in Guinea was 35.8% in 1999, 38.9% in 2005, 46.3% in 2012 and 62.3% in 2016.. NMR was 51.8 per 1000 live births in 1999, 54.8 in 2005 and 40.4 in 2012. The reduction of NMR especially in 2012 might be partly explained by better coverage of SBA as documented in prior studies [16–18]. Absolute and relative inequalities with respect to SBA existed in all four surveys in Guinea from 1999 to 2016. Moreover, absolute, and relative socioeconomic, gender and geographic inequalities in NMR were seen between 1999 and 2012 with higher risks among male neonates and from disadvantaged subpopulations.

It is documented that educated and wealthier women have higher odds of maternal health care utilization [37]. Educated women have increased awareness of health problems and available healthcare facilities [38]. In addition, educated women are more empowered which again gives confidence and autonomy to use health care [39, 40]. Different scholars have revealed that educated women have higher confidence to make their own health decisions and better knowledge about the benefits of utilizing health care than non-educated women [41–43].

Pro-rich inequalities in SBA in Guinea are consistent with previous studies in Bangladesh [44–46]. Gender-based variations of NMR observed in Guinea were comparable with prior studies in Burundi and Angola [31, 47]. Possible reasons could be due to inability to afford costs related to childbirth among households with lower socio-economic status [46]. Lack of financial resources is a common barrier to the utilization of healthcare including SBA among the poor, even when direct costs for care are exempted or care is provided free of cost [48]. Several countries in SSA have policies to lower/exempt direct out-of-pocket (OOP) costs to expand and promote using maternal health care [48, 49]. Direct OOP costs related to maternity care include all formal, official fees charged for birth care, hospitalization and necessary medicine and supplies. Besides direct financial expenses, there may be extra or unforeseen costs of care-seeking like loss of working time and wages [48]. Those unforeseen costs are difficult to measure as they differ based on revenue and occupation status and may be subject to periodic/seasonal variation as well [48]. Indirect costs and informal fees of care-seeking can sometimes exceed direct OOP costs [50]. This indicates that, in addition to direct costs, indirect costs also need policy attention to increase maternal health care and equitable health care use [48]. Wealth-driven inequality in SBA coverage, decreasing by complex measures (PAR, PAF), especially from 2012 to 2016 might be due to the fact that complex measures take all five wealth subgroups into consideration, unlike simple measures (D, R) [33]. There is, of course, more percentage points to gain when the SBA coverage is low and will become lesser in groups with already higher SBA coverage.

Profound pro-urban inequalities in SBA and NMR are comparable with prior studies in Angola and Burundi [31, 47]. Urban-rural differences in SBA have been reported in previous studies in Ethiopia and Bangladesh [46, 51]. This may be partly due to lack of transport, poor access, costs, and distance to health facilities [52, 53]. Studies in Sudan and India showed that place of residence had effect on the uptake of maternal health care [54, 55]. Women residing in rural areas in Tanzania most had transport difficulties to health facilities and getting medical treatment [56]. The narrowing of urban-rural inequality in SBA during the years in Guinea is a sign of the country’s dedication to strengthen RMNCH care and utilization in remoter parts of the country post-Ebola. The national health Plan 2015–2024 outlines the government’s goal of achieving a set of RMNCH indicator goals in each of the eight administrative regions by 2024.

Male babies appeared to have higher neonatal mortality, which may be due to intrauterine growth restriction, respiratory insufficiency, or prematurity, comparable with a prior study in Burundi [31]. Moreover, the role of gender-associated genetic and endocrine differences are also hypothesized for this variation [31, 57].

Important geographic-based disparities in SBA and NMR were comparable with studies in Tanzania, Benin, Angola, and Burundi [31, 47, 58–60]. Number of health personnel and health facilities, availability of essential medicines or supplies and the quality of care may explain differences in SBA coverage across regions [60]. In Guinea, most doctors, nurses, and midwives work in city areas, despite often being officially deployed somewhere else. There are not any functioning accountability systems to ensure that health workers stay where they have been officially deployed. The predominantly urban job desire of health workers is not surprising. WHO has recommended four critical interventions to improve attraction, recruitment and retention of health workers in remote and rural areas including education, regulation, financial incentives, and professional and personal support [61]. If implemented, these would go a long way towards reducing geographic-based inequalities in SBA and NMR in other health outcomes.

## Strengths and limitations

Magnitude of and trends in SBA and NMR inequalities were examined using the WHO HEAT software that stores re-analyzed DHS and MICS data carried out by health disparity experts. The extensive analytic procedures allow for comparison of findings and strengthen the quality of our study. Additionally, application of absolute and relative, as well as simple and complex summary measures, in a single study has benefits for policy makers and programmers to view the magnitude of the problem from different perspectives. Our study also has limitations. The prescriptive analytic strategies did not allow us to explore other social, demographic, or cultural factors related to inequalities in SBA.

## Conclusion

Inequalities in SBA coverage in Guinea favored women in the two richest wealth quintiles, urban residents, and women from regions such as Conakry. Substantial inequality in NMR was observed between 1999 and 2012 with more concentration on male neonates and those from mothers who were uneducated, from the two poorest wealth quintiles, rural residents, and subnational regions such as Faranah (1999 and 2005) and Kankan (2012). SBA inequalities continued throughout the four surveys in a relatively decreasing pattern even though these varied based on summary measures.

To reduce NMR, we recommend that the Guinea government and other stakeholders working in the maternal and neonatal health sector prioritize disadvantaged population groups who lack access to essential maternal health care including SBA. Empowering women through education and economic opportunities could help ensure more equitable access to SBA in order to reduce NMR. Additionally, pro-rural SBA and other neonatal health improvement interventions and giving priority to women from less advanced regions or with low SBA coverage and higher NMRs are recommended to ensure that socially disadvantaged subpopulations are not left behind.

## Data Availability

The datasets generated and/or analyzed during the current study are available in the WHO’s HEAT version 3.1 [https://www.who.int/gho/health_equity/assessment_toolkit/en/].

## Abbreviations

D: Difference
DHS: Demographic and Health Survey
EA: Enumeration Area
HEAT: Health Equity Assessment Toolkit
MICS: Multiple Indicator Cluster Survey
MMR: Maternal mortality ratio
NMR: Neonatal Mortality rate
OOP: Out of Pocket
PAF: Population Attributable Fraction
PAR: Population Attributable Risk
R: Ratio
SBA: Skill Birth Attendant
SDGs: Sustainable Development Goals
SDGs: Sustainable Development Goals
UI: Uncertainty Interval
WHO: World Health Organization
